# Exosomal circRNAs: new players in colorectal cancer

**DOI:** 10.1186/s12935-021-02112-6

**Published:** 2021-09-14

**Authors:** Faezeh Vakhshiteh, Shokoufeh Hassani, Navid Momenifar, Fatemeh Pakdaman

**Affiliations:** 1 Nanotechnology Research Centre, Faculty of Pharmacy, Tehran University of Medical, Sciences, Tehran, Iran; 2 Toxicology and Diseases Group (TDG), Pharmaceutical Sciences Research Center (PSRC), The Institute of Pharmaceutical Sciences (TIPS), Tehran University of Medical Sciences, Tehran, Iran; 3 Department of Toxicology and Pharmacology, School of Pharmacy, Tehran University of Medical Sciences, Tehran, Iran; 4 Human and Animal Cell Bank, Iranian Biological Resource Center (IBRC), ACECR, Tehran, Iran; 5 Department of Genetics, Faculty of Science, University of Shahrekord, Shahrekord, Iran

**Keywords:** Exosome, circRNA, Colorectal cancer

## Abstract

Colorectal cancer (CRC) is one of the most malignant cancer types, characterized by elevated mortality rate and treatment resistance. Despite the progress achieved in the explanation of the molecular basis of the disease as well as introducing potential biomarkers in the clinical practice, further investigation is essential to identify innovative molecules that contribute to colorectal carcinogenesis. Circular RNAs (circRNAs) are a novel and unexplored RNA type, associated with various human pathological conditions. Recently, circRNAs have been identified to be enriched and stable in exosomes and can exert their functions when exosomes reach neighboring or distant cells. Increasing evidence indicates that these so called exosomal circRNAs (exo-circRNAs) act as signaling molecules to regulate cancer proliferation, metastasis, and sensitivity to radio- and chemotherapy. This review aims to discuss the latest progress in exo-circRNAs studies in CRC with an emphasis on their potential as promising diagnostic molecular markers and therapeutic targets.

## Introduction

Circular RNAs (circRNAs) are an exclusive class of long non-coding RNAs, which are produced by a covalent linkage via back-splicing of linear RNA [1]. With the development of next generation sequencing and bioinformatics in the twenty-first century, the abundance and diversity of circRNAs was identified. Unlike linear RNAs, circular RNAs have a special circular covalently bonded structure, which give them a higher tolerance to RNase degradation [2]. Besides from high abundance and stability, circRNAs show cell type- or tissue-specific expression patterns [3]. These features make the circRNAs as unique molecular markers in some human diseases, including cancer.

One of the most revolutionary contributions to cell biology was the discovery of exosomes; a class of extracellular vesicles secreted by almost all cell types that circulate in bodily fluids such as blood, urine, and saliva [4]. Exosomal contents composed of different proteins, lipids, and nucleic acids, long noncoding RNAs, as well as circRNAs [5, 6]. CircRNAs can be sorted into exosomes along with other molecules such as nucleic acids, lipid and proteins. The exosomes are then released by the parent cells into bodily fluid, through which exosomal circRNAs (exo-circRNAs) initiate their circulation and their biological functions. Particularly, exo-circRNAs were shown to play an important role in cancer initiation, progression, and therapy resistance [7–9]. In this review, we summarize the biogenesis, characteristics, and biological functions of exo-circRNAs and then discuss the current progression of exo-circRNA in colorectal cancer (CRC) and its implication as biomarker in addition to their therapeutic potential.

## Colorectal cancer

CRC is one of the most aggressive cancer types considered as the fourth leading cause of cancer deaths globally. The disease public health burden is anticipated to rise by 60% to more than 2.2 million new cases by 2030 [10]. The poor prognosis of late-stage tumors is associated to a number of factors, such as the high potential of metastasis, difficulty in early detection, and the resistance to conventional therapies. While most diagnoses occur in developed counties, less developed areas have shown poorer survival rate and the incidence and mortality rates of CRC in some regions are continually increasing [11]. Therefore, highly sensitive and convenient biomarkers are essential for diagnosis of CRC.

## Biogenesis of exosomes

**Fig. 1:**
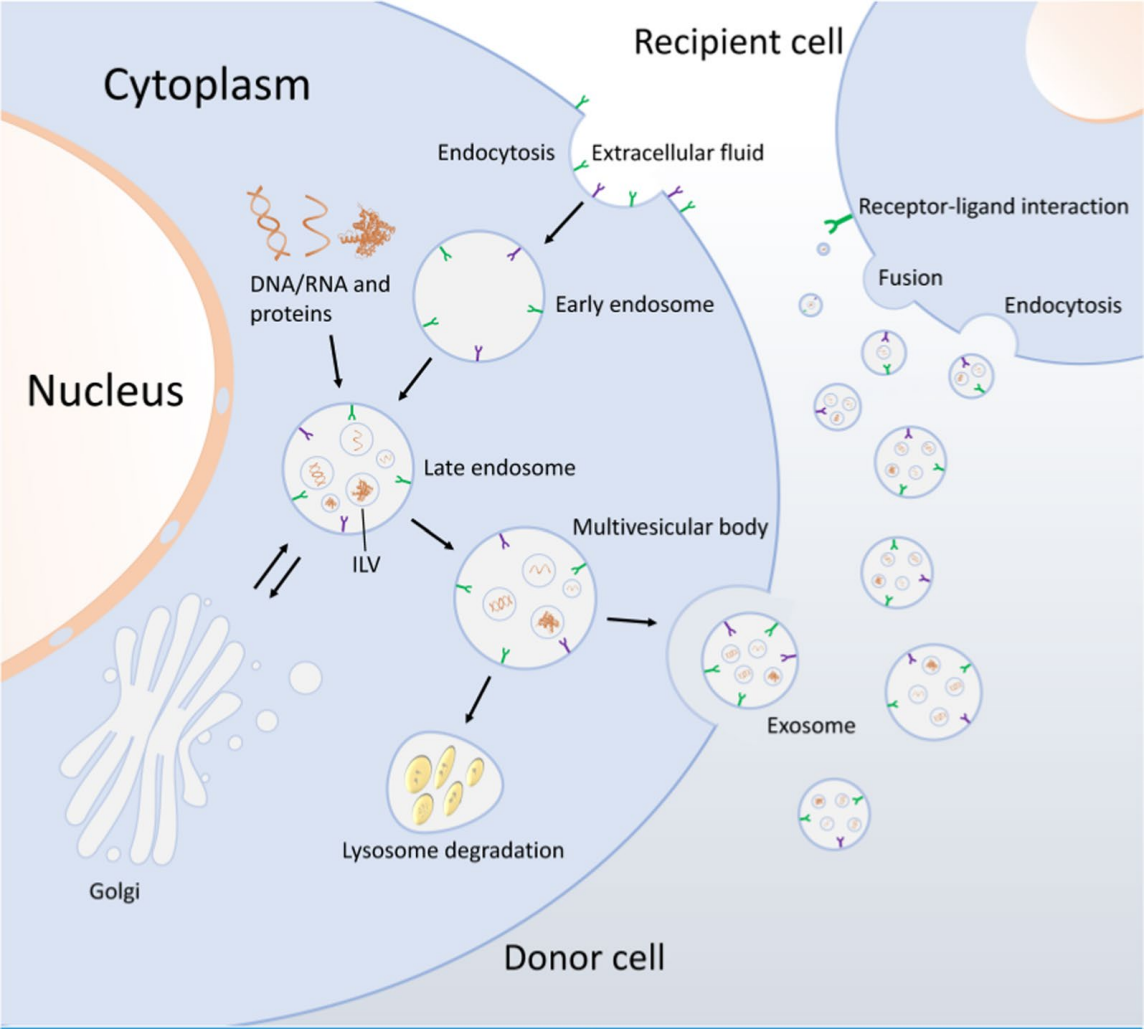
Biogenesis of exosomes. The biogenesis of exosomes consist of endocytosis, MVBs formation, and exosome secretion into the extracellular space through merging with the plasma membrane

Oncogenic molecules could be transported from cancer cells to the surrounding environment via extracellular vesicles or exosomes [12, 13]. Exosomes are extracellular vesicles that are generated in the endosomal compartment and secreted by almost all cell types [4]. These tiny vesicles are packed with the intercellular materials, which then secreted and affect several physiological and pathological functions in the recipient cells [14]. The biogenesis of exosome begins within the endosomal compartments. Early endosomes are turned into late endosomes to form multivesicular bodies (MVBs) through various routes. In this procedure, endosomal membrane invaginates to produce intra-luminal vesicles (ILVs) within its own lumen. Subsequently, MVBs either merge with lysosomes for degradation or secrete ILVs into the extracellular space through merging with the plasma membrane [15] (Fig. 1).

The data regarding the exosomal composition is available in databanks including Exocarta (http://www.exocarta.org), Vesiclepedia (http://microvesicles.org), and Evpedia (http://evpedia.info) that are updated regularly [16]. Exosomes encompass an intricate combination of proteins such as membrane fusion proteins, major histocompatibility complex (MHC) proteins, heat shock proteins, and cytoskeleton proteins [17]. Furthermore, a variaty of cell–specific proteins has been described, which can differ based on the pathophysiological states. In addition, numerous forms of nucleic acids such as mRNAs, miRNAs, lncRNAs, ribosomal RNAs, small nucleolar RNAs, and circRNAs have been reported in exosomes [5, 6]. The RNA types are transported from producer cells via exosomes and can generate some effects in the recipient cells. The late investigations authenticated that circRNAs are stable in exosomes and can exert their effects after exosomes are released from the produer cells into the surrounding environment [18]. In addition, as indicated in Table 1, further studies confirmed the application of exosomal circRNA, referred to as exo-circRNA, as biomarkers for various cancers [19].

**Table 1:** The prospective diagnostic and therapeutic biomarkers of exo-circRNAs related to CRC

circRNA	Role/Function	Regulation	Sample	References
*circKLDHC10*	Biomarker	↑	Serum exosome	[19]
*hsa_circ_0004771*	Biomarker	**↑**	Cell line; seum exosome	[38]
*hsa_circ_0101802*	Biomarker	**↑**	Serum exosome	[39]
*hsa_circ_0000338*	Drug resistance	**↑**	Cell line; serum exosome	[42]
*circ_0032883*	Drug resistance	**↑**	Cell line exosome	[42]
*circ_0066629*	Drug resistance	**↑**	Cell line exosome	[42]
*ciRS-122*	Drug resistance	**↑**	Cell line exosome	[43]
*circ-FBXW7*	Drug resistance	↓	Cell line; serum exosome	[44]
*circ-PACRGL*	Enhanced proliferation and invasion of cancer cells via miR-142-3p/miR-506-3p sponge to support the expression of TGF-β1	↑	Cell line exosome	[45]
*circ-133*	Biomarker/enhanced metastasis via miR-133a/GEF-H1/RhoA axis	↑	Serum exosome	[46]
*hsa_circ_0008558*	Enhanced metastasis via modifying the maturation and allocation of miR-17	↑	Cell line exosome	[47]
*circ_IFT80*	Enhanced proliferation and inhibited radiosensitivity by regulating miR-296-5p/MSI1 axis	↑	Cell line; serum exosome	[48, 49]
*hsa_circ_0000677*	Biomarker/enhanced metastasis and stemness properties possibly by upregulation of circ_0000677 via Wnt pathway	↑	Cell line exosome	[50]
*circFAT1*and *circRTN4*	Downregulation of circFAT1 and circRTN4 in mutant cells, suggesting a potential involvement of circRNAs in oncogenesis via increased exporting of the circRNAs to exosomes	↑	KRAS mutant cell line exosome	[51]

## CircRNA biogenesis and function

CircRNAs are recognized as a member of diverse family of endogenous noncoding RNAs, which demonstrate a new research hotspot in the field of cancer biology [20]. circRNAs were primary considered as transcriptional junk or by-products of non-standard splicing of RNA [21]. Later, by progress in bioinformatics and RNA sequencing technologies, the abundance and diversity of circRNAs was approved, and the particular expression patterns of circRNAs were demonstrated in pathological conditions [2].

**Fig. 2:**
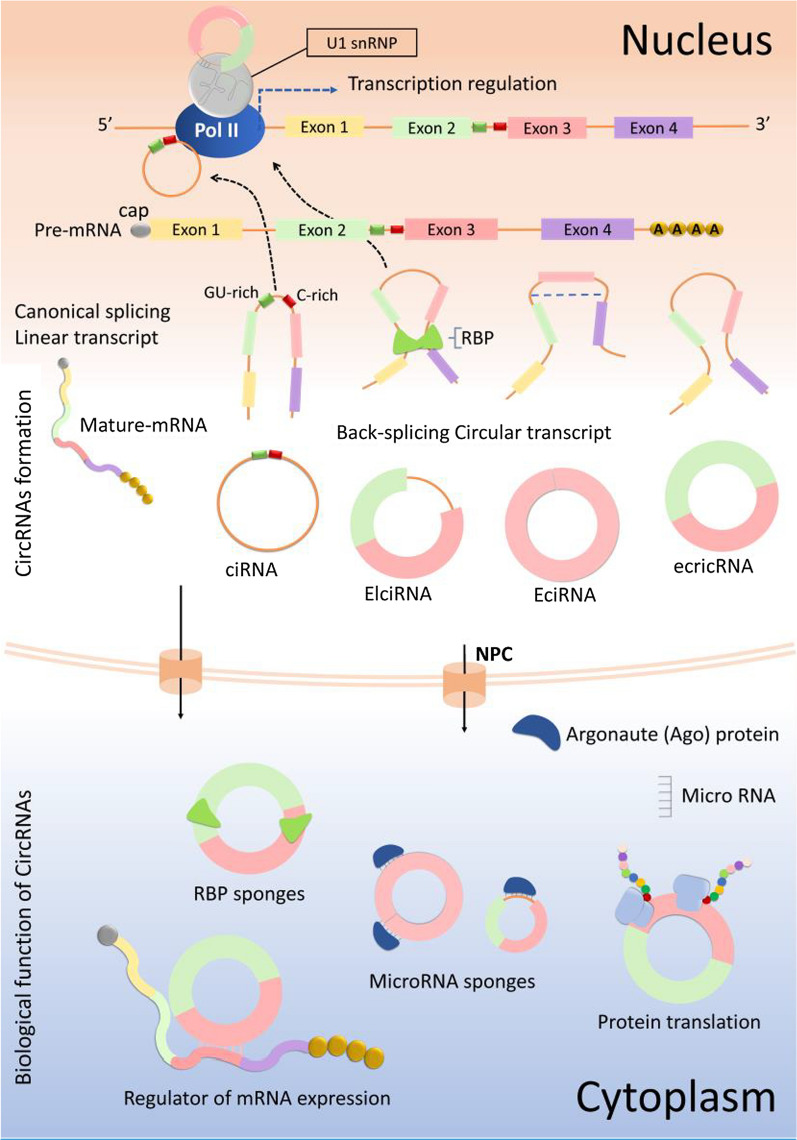
Biogenesis and function of circRNAs. CircRNAs are produced by back-splicing, wrap up into exosomes, and secreted into the extracellular environment. The potential functions of circRNAs in multiple biological processes have been identified, including miRNA sponges, alternative splicing, and transcriptional or post-transcriptional gene regulation. CircRNAs are mainly act as miRNA sponge to control expression of target genes via preventing the of miRNA activity. CircRNAs also adjust gene expression via attachment to miRNAs and releasing them from their target genes. Some circRNAs that exhibit attachment sites for RPB may function as protein sponges besides from performing as miRNA sponges

CircRNAs vary from structure of lncRNAs in their 3’ and 5’ terminus, which are covalently attached [1]. The structure of circRNAs, unlike their linear transcripts, is closed rings without tails in the 5′–3′ terminus. This attachment occurs at a site flanked by recognized splice indicators and in order to obtain a circRNA a splice donor has to be attached to an upstream splice acceptor (Fig. 2). This phenomenon is known as “back-splice” and is unique for circRNAs and vary from the normal splicing form in a way that splice donor is fused to a downstream splice acceptor [22]. CircRNAs are commonly categorized as three forms: exonic circRNAs, intronic circRNAs, and exonic-intronic circRNAs. While exonic circRNAs present in cytoplasm, the intronic and exonic-intronic circRNAs are mostly located in the cell nucleus [23, 24].

Currently, numerous circRNAs have been discovered to adjust gene expression at transcriptional, posttranscriptional, and translational levels [25]. The circRNA-mediated regulation mechanisms have been demonstrated by various studies (Fig. 2). So far, circRNAs have been revealed to perform as miRNA sponges, alternative-splicing regulators, binding to RNA-binding proteins (RBPs) as well as encoding proteins. The most remarkable of the mentioned mechanisms of action of circRNAs is that they could act as miRNA sponges [26]. CircRNAs can inhibit the attachment of miRNAs with the 3’ UTR of a particular gene by attachment to miRNAs, ultimately regulating the gene expression by triggering mRNAs cleavage or mRNA translation suppression. For example, circNRIP1 can acting as a sponge of microRNA-149-5p and subsequently block its effects in cancers [27]. Various circRNAs harbor binding sites for multiple miRNAs and regulate them all at once and these evolutionarily conserved binding sites guarantee the deficiency of targets [25]. Additionally, circRNAs are capable of accumulating in cells and preserve their role for a relatively longer period owing to the great stability. Furthermore, particular circRNAs have numerous sites for RBPs, so that they can block the activity of RBPs by performing as sponge of proteins [28, 29]. For example, *circARSP91* cooperates with AR-ADAR1, thus suppressing tumor growth in hepatocellular carcinoma [30]. Furthermore, circRNAs are regarded to function as protein scaffolds through harboring binding sites for the protein assembly, which may consequently generate great complexes of proteins. For instance, circ-forkhead box O3 (Foxo3) might form a circ-Foxo3-p21-CDK2 ternary complex by interacting with CDK2 and p21, which acts as CDK2 blocker [31].

It is becoming evident that deregulated expressions of certain circRNAs participate in the initiation and development of cancer. Particularly, circRNAs play vital roles in cancer proliferation, metastasis, and treatment resistance [7–9]. Different circRNAs have been demonstrated to be expressed abnormally in CRCs. Bioinformatical analysis of circRNAs expression in human have indicated that most of circRNAs are noticeably downregulated in CRCs [10]. RNA-seq records demonstrated that the percentage of circRNAs to their related linear transcripts was frequently declined in CRC models in comparison to the corresponding normal colon tissues and considerably poorer in related cancerous cell lines, which seems to be independent from expression amount of circRNAs or linear RNAs elevation or decline in cancer patients in comparison with noncancerous samples [32]. These data make circRNAs as promising prognosis biomarkers and therapeutic goals in cancer therapy [33, 34].

Latest investigations demonstrated that circRNAs are highly enriched in exosomes, maybe because of the protective properties of exosomes or particular sequence structures and/or its protein associates [19, 35]. Due to the double-layered membrane of exosomes, the payload in being protected from being cleared or degraded [36]. The nano-scale size of exosomes also supports to extend the circulation time and improves the biological activities of circRNAs [37]. Previous studies have demonstrated that exo-circRNAs may have significant regulatory advantages; hence the practice of exosomes together with circRNAs could upsurge the possible clinical implication of these molecules as biomarkers in cancer diagnosis and prognosis. Interestingly, exosomal circRNAs are abundant and stable, particularly in those exosomes derived from tumors compared to bare circRNAs that are released from cells. Based on the published reports, exo-circRNAs were enriched in exosomes in comparison to the levels of circRNA in parental cells by at least twofold. It was also indicated that in exosomes the amount of circRNA to linear RNA level was almost sixfold superior compared to that of the cells, demonstrating that circRNAs were abundantly incorporated into exosomes compared to that of linear RNA [19]. Consequently, the high amount of stable exo-circRNAs represents an innovative class of RNA species that may differentiate healthy individuals from patients with cancer demonstrating its substantial translational capability as a circulating biomarker for cancer diagnosis. Despite the progress in this field, the role of exo-circRNAs in CRC progression has not been clearly elucidated. Therefore, this review aimed to evaluating the current advances in exosomal circRNA study in CRC and to summarize the present implication of circRNAs of exosomes in the initiation, development, and treatment resistance in CRC, in addition to their potential clinical significance as biomarkers and therapeutic targets.

## Exo-circRNAs serve as potential biomarkers

The existence of plentiful circRNAs in exosomes was demonstrated by Li et al. [19]. The group identified more than 1000 circRNAs in human serum exosomes. They proposed that serum exo-circRNA may differentiate patients with cancer from healthy individuals, showing its important translational prospective as circulating biomarkers for diagnosis of cancer. According to the findings of this study, expression levels of *circ-KLDHC10* were significantly elevated in CRC serum exosomes compared to those in normal individuals.

Pan et al*.* reported that the circulating exosomal *has_circ_0004771* was considerably upregulated in CRC patients with a sensitivity of 81% and a specificity of 80%. The exosomal *has_circ_0004771*, which was the most enriched circRNAs among the uppermost differentially expressed circRNAs, was selected according to the results of Gene Expression Omnibus (GEO) dataset analysis in a total of 170 patients and 45 healthy individuals joined in this study. Thus, the *has_circ_0004771* has the potential to be practiced as a new potential biomarker for early diagnosis of CRC [38].

Xie et al*.* demonstrated the diagnostic value of exosomal *hsa_circ_0101802* (*exo-circ-PNN*) in CRC. According to this study, the clinical relevance of serum exo-circRNAs in CRC cases was analyzed by circRNAs sequencing (RNA-seq) in a total RNA sample from 50 CRC patients and 50 normal individuals. It has been found that the levels of *exo-circ-PNN* in serum were considerably elevated in CRC patients compared to the normal individuals. Therefore, it may be a promising biomarker for the detection of CRC and may contribute to the pathogenesis of CRC [39]. Similarly, Li et al. stated that serum *circKLDHC10* could differentiate patients with CRC from normal individuals, demonstrating its substantial potential as a circulating noninvasive biomarker for CRC diagnosis.

## Therapeutic potential of exo-circRNAs

Quite a few investigations have revealed the association between circRNAs and signaling pathways that are linked to tumor initiation and aggressive features of CRC (Table 1). Generally, upregulated circRNAs act as oncogenes in CRC and stimulate cell proliferation and metastasis while preventing cell cycle arrest and programmed death. Almost 1215 circRNAs have been recognized in human serum exosomes, which can penetrate to the recipient cells and instead bind to specific miRNAs and/or RBPs, leading to generation of functional complexes with potential therapeutic applications in cancer therapy [10].

The standard treatment in CRC is surgery combined with chemotherapy; however, the chemo-resistance has become a burden in treating the disease effectively. The contribution of circRNAs to chemo-resistance in some types of cancers has been described [40]. However, the role of exo-circRNAs in CRC remains largely elusive. Previous studies have demonstrated that exosomes can relocate nucleic acids from chemo-resistant tumor cells into drug-sensitive cells to develop the resistance ability in cancers [41]. To investigate the crosstalk between circRNAs in exosomes and drug-resistance in CRC, Hon et al. showed that *has_circ_0032883*, *has_circ_0000338*, and *has_circ_0066629* were upregulated in exosomes derived from drug-resistant cells [42]. Furthermore, selective transfer of exosomal *hsa_circ_0000338* via drug-resistant tumor cells into co-cultured drug-sensitive cells indicated superior viability towards drug treatment, suggesting that exosomal *hsa_circ_0000338* might contribute to increase drug resistance of acceptor cells.

Recent studies have indicated that expression amount of *exo-ciRS-122* in serum is positively linked with chemoresistance. An in vitro and in vivo investigations confirmed that oxaliplatin-resistant cells derived exosomes could convey *ciRS-122* to chemo-sensitive cells, in which glycolysis and drug resistance were boosted by shrinking *miR-122* and upregulating *PKM2* [43]. Alternatively, the suppression of *ciRS-122* inhibited glycolysis and overturned the resistance to oxaliplatin in colorectal cancer. Another study has confirmed that *circ-FBXW7* was reduced in oxaliplatin-resistant CRC patients and cells [44]. Subsequent cell culture and animal studies revealed that *exo-circ-FBXW7* could modulate resistance to oxaliplatin by directly binding to miR-18b-5p, leading to an increase in apoptosis, inhibition of epithelial-mesenchymal transition, and suppression of oxaliplatin efflux. In summary, exosomes contribute to mediating chemo-resistance from drug-resistant cells to drug-sensitive cells by transporting circRNA. Therefore, exosomal circRNA could function as a promising new target for the management of drug-resistant CRC.

Transforming growth factor-beta (TGF-β) is a cytokine which is linked to initiation, development, and metastasis of tumors and function as a gatekeeper of the immune system and modulator of quite a few signaling pathways in tumors. Shang et al*.* showed that exosomal *circ-PACRGL* contributes to ontogenesis by promoting growth and progress of CRC. The circ-PACRGL performed as *miR-142-3p/miR-506-3p* sponge to support the expression of *TGF-β1* [45]. The *circ-PACRGL* also promoted the N1 to N2 neutrophils differentiation by modulating miR-142-3p/miR-506-3p-TGF-β1 axis, which provides a novel justification for the exploration of strategies for CRC therapy.

Hypoxic condition is an intrinsic feature of the fast growing solid tumor. Due to the heterogeneous oxygen partial pressure in solid tumors, intercellular heterogeneity of cancer energy metabolism occurs, leading to variances in storage of energy in normoxic and hypoxic cells; hence, various metastasis capacities revealed. Yang et al*.* demonstrated that exosomal c*irc-133* is enriched in CRC patients’ plasma samples and improved with the disease progression [46]. According to this study, hypoxia-derived *exo-circ-133* delivery into normoxic tumor cells supported cancer metastasis via miR-133a/GEF-H1/RhoA axis. Meanwhile, blocking of *circ-133* inhibited cancer metastasis. Thus, *Circ-133* is anticipated to be a novel noninvasive biomarker to follow cancer progress and can be regarded as a new target for therapeutic strategies.

It has been recognized that tumor cells with great metastatic characteristics could associate with surrounding cells via exosomes to make the primary microenvironment easier for metastasis-initiation. Han et al*.* demonstrated that *hsa_circ_0008558*, referred to as *circLONP2* performed as an important metastasis-initiating player in CRC progression via modifying the maturation and allocation of *miR-17*, which leads to dissemination of metastasis-initiating capacity and speeding up the metastasis formation [47]. It was further investigated that *circLONP2* downstream targets engaged in this process by directly being packed into exosomes and conveyed to neighboring cells to enhance their aggressiveness.

Other studies demonstrated that *circ_IFT80* contributed to the tumorigenesis and supressed radiosensitivity in CRC by regulating miR-296-5p/MSI1 axis [48, 49]. The *circ_IFT80* performed as *miR-296-5p* sponge, which reversed the effects of this circRNA on cancer cells proliferation and radiosensitivity. Moreover, *miR-296-5p* repressed tumor growth and supported radiosensitivity by downregulating MSI1, a direct target of *miR-296-5p*. Therefore, it could be considerd as a potential prognostic predictor and/or new therapeutic target in CRC therapy. Similarly, exosomes from CD133^+^ cells carrying *hsa_circ_0000677* (*circ-ABCC1*) mediate cell stemness and metastasis in CRC. Zhao et al*.* explored that exosomes derived from CD133^+^ cells promoted cell stemness, sphere formation, and metastasis possibly by upregulation of *circ-ABCC1* via Wnt pathway [50].

Dou et al*.* detected circRNAs in secreted exosomes from wild type/mutant KRAS colon cancer cell lines [51]. Importantly, It was shown that circRNA abundance was down-regulated in mutant KRAS cell lines compared to wild type cells, suggesting a potential involvement of circRNAs in oncogenesis. Based on this study, one possibility is that down-regulation of circRNAs in KRAS mutant cells is caused by their increased exporting to exosomes.

## Conclusion and future prospective

The association of exosomal circRNAs with cancer has turned into an interesting research field. circRNAs have shown to contribute to cancer progression by modulating cellular growth, invasion, migration, metastasis, and drug resistance. The combined application of exosomes and circRNAs can enhance the potential use of exo-circRNAs as diagnosis and prognosis biomarkers for cancer. Based on the observations of this study, a number of exosomal circRNAs were implicated in CRC. Some of these circRNAs were shown to play role as biomarkers and may provide a novel pathway for cancer diagnosis. Some other exo-circRNAs were indicated to promote proliferation, metastasis, radioresistance, drug resistance, and stemness properties and therefore, might have potential for monitoring disease progression or recurrence. The typical features of exo-circRNAs, such as stability, sensitivity, and specificity allow their use in clinical practice. Exosomal circRNA can transfer biological materials to target cells while protecting their cargo from clearance. Despite the encouraging progress in this field, a number of issues need to be addressed. While various investigations revealed that exosomal circRNAs maintain biological activity as miRNA sponges, it remains largely unknown whether they can perform as protein scaffolds or templates for protein translation. Due to lack of standardized method for collecting, processing and isolating exosome samples, adequate standards for exosome isolation and characterization need to be established to bring this exciting field a step closer to clinical reality. Furthermore, the exact mechanism of how circRNAs are enriched during exosome biogenesis is unknown. In other words, it is not clear whether circRNAs may be passively included in exosomes during biogenesis or they might be actively transferred from the cytoplasm into exosomes. Considering the potential application of exo-circRNAs as cancer biomarkers, much work is needed to compare circRNA expression profiles between cancer cells and their exosomes in order to assure whether signatures of exo-circRNAs can reflect those of original cancer cells. Moreover, investigations with larger cohorts are warranted to demonstrate that exo-circRNAs are appropriate noninvasive biomarkers in clinical settings. Finally, circRNAs are difficult to be detected in exosomes due to their low abundance, which necessitate advanced approaches and equipment. Therefore, it is important to develop and use appropriate methods and techniques to elucidate the molecular mechanisms and regulatory networks of exo-circRNAs. As exosomes naturally transfer various cargoes, there is a possibility of taking advantage of exosomes to deliver therapeutic molecules to cancer cells. Loading exosomes with therapeutic circRNAs may demonstrate a practical approach for cancer therapy. With regard to promoting effect of many circRNAs in cancer, exosomes carrying drugs, such as specially designed small interfering RNAs (siRNAs) that target specific circRNAs, can help lower the expression of negative circRNAs in cancer cells. Even with their promising prospects, there is still a long way to go to reach the goal of developing exo-circRNA-based cancer diagnostic and therapeutic strategies.

## Data Availability

Not applicable.
